# Expression of insulin receptor (IR) A and B isoforms, IGF-IR, and IR/IGF-IR hybrid receptors in vascular smooth muscle cells and their role in cell migration in atherosclerosis

**DOI:** 10.1186/s12933-016-0477-3

**Published:** 2016-12-01

**Authors:** N. Beneit, C. E. Fernández-García, J. L. Martín-Ventura, L. Perdomo, Ó. Escribano, J. B. Michel, G. García-Gómez, S. Fernández, S. Díaz-Castroverde, J. Egido, A. Gómez-Hernández, M. Benito

**Affiliations:** 1 Biochemistry and Molecular Biology II Department, School of Pharmacy, Complutense University of Madrid, Plaza Ramón y Cajal s/n, 28040 Madrid, Spain; 2 Health Research Institute of San Carlos Clinic Hospital (IdISSC), Madrid, Spain; 3 CIBER of Diabetes and Associated Metabolic Diseases (CIBERDEM), Barcelona, Spain; 4 Vascular Research Lab, IIS-Fundación Jiménez Diaz-Autonoma University, Madrid, Spain; 5 Inserm, U698, Universite Paris 7, CHU X-Bichat, Paris, France

**Keywords:** Atherosclerosis, Insulin receptor, Migration, Vascular smooth muscle cells

## Abstract

**Background:**

Abnormal proliferation and migration of vascular smooth muscle cells (VSMCs) is a major contributor to the development of atherosclerotic process. In a previous work, we demonstrated that the insulin receptor isoform A (IRA) and its association with the insulin-like growth factor-I receptor (IGF-IR) confer a proliferative advantage to VSMCs. However, the role of IR and IGF-IR in VSMC migration remains poorly understood.

**Methods:**

Wound healing assays were performed in VSMCs bearing IR (IRLoxP^+/+^ VSMCs), or not (IR^−/−^ VSMCs), expressing IRA (IRA VSMCs) or expressing IRB (IRB VSMCs). To study the role of IR isoforms and IGF-IR in experimental atherosclerosis, we used ApoE^−/−^ mice at 8, 12, 18 and 24 weeks of age. Finally, we analyzed the mRNA expression of total IR, IRB isoform, IGF-IR and IGFs by qRT-PCR in the medial layer of human aortas.

**Results:**

IGF-I strongly induced migration of the four cell lines through IGF-IR. In contrast, insulin and IGF-II only caused a significant increase of IRA VSMC migration which might be favored by the formation of IRA/IGF-IR receptors. Additionally, a specific IGF-IR inhibitor, picropodophyllin, completely abolished insulin- and IGF-II-induced migration in IRB, but not in IRA VSMCs. A significant increase of IRA and IGF-IR, and VSMC migration were observed in fibrous plaques from 24-week-old ApoE^−/−^ mice. Finally, we observed a marked increase of IGF-IR, IGF-I and IGF-II in media from fatty streaks as compared with both healthy aortas and fibrolipidic lesions, favoring the ability of medial VSMCs to migrate into the intima.

**Conclusions:**

Our data suggest that overexpression of IGF-IR or IRA isoform, as homodimers or as part of IRA/IGF-IR hybrid receptors, confers a stronger migratory capability to VSMCs as might occur in early stages of atherosclerotic process.

**Electronic supplementary material:**

The online version of this article (doi:10.1186/s12933-016-0477-3) contains supplementary material, which is available to authorized users.

## Background

Atherosclerosis is the leading cause of mortality worldwide. The progression of vascular lesions from early fatty streaks to more advanced plaques is a complex process [1] where vascular smooth muscle cells (VSMCs) plays a main role. The presence of a large number of intimal VSMCs has been taken as evidence that VSMC migration from the media plays an important role in early stages of atherogenesis [2]. These VSMCs that migrated into the intima exhibit an abnormally increased proliferation and extracellular matrix production, leading to the formation of the fibrous cap in atherosclerotic lesions [3]. Growth factors, including insulin-like growth factors (IGFs), have been implicated in the regulation of VSMC migration [4, 5].

The insulin and IGFs (IGF-I and IGF-II) signaling is mediated by hormone interaction with the insulin receptor (IR) and the IGF-I receptor (IGF-IR) which are members of subclass II of the tyrosine kinase receptor super-family [6, 7]. Both receptors are expressed at the cellular surface as preformed disulfide-linked dimers in α2β2 configuration. The extracellular α subunit of each hemireceptor contains the ligand binding sites, while β subunits include a large cytoplasmic region with tyrosine kinase activity [8]. Because of the high degree of homology of the two receptors, hybrid receptors formed by an IR αβ-hemireceptor and an IGF-IR αβ-hemireceptor are also found in cells co-expressing IR and IGF-IR [9–11].

Alternative splicing of the IR gene gives rise two isoforms, IRA and IRB [12], whereas there is only a single isoform of IGF-IR. Indeed, IRB differs from IRA by the inclusion of exon 11 which encodes a 12-amino acid sequence at the C-terminus of the IR α-subunit. The IR isoforms show different functional features. Although both isoforms have similar affinity for insulin, IRA exhibits a higher affinity for IGFs, especially for IGF-II [13], as well as a greater internalization and recycling rate than IRB [14]. Because of these differences, IRB is preferentially associated with metabolic and differentiating signals. Conversely, IRA mainly favors cell growth, proliferation, and survival. In 32D cells, IRA induces mitogenic and antiapoptotic signals in response to IGF-II, whereas IRB tends to send differentiation signals [15]. In mouse beta cell lines, IRA confers a stronger proliferative capacity favoring the mitogenic effects of IGF-I and increasing glucose uptake [16], and it also might provide an explanation for the beta cell hyperplasia induced by liver insulin resistance in iLIRKO mice [17]. Additionally, long-term AAV-mediated hepatic expression of IRA, but not IRB, improves glucose homeostasis in iLIRKO mice, precluding beta cell mass expansion and, therefore, avoiding the final beta cell failure [18]. IRA is also more efficient than IRB at increasing glycogen synthesis, glycogen synthase activity and glycogen storage in hepatocytes and in vivo in the liver [19]. Furthermore, IRA has been reported to be the predominant IR isoform expressed in cancer cells, such as in a variety of carcinomas or breast cancer cell lines [11, 13, 20].

A further understanding of the molecular mechanisms involved in early atherosclerosis is critical for identifying strategies to limit disease progression before it leads to clinical consequences. In this regard, we previously demonstrated that IRA, but not IRB, confers a proliferative advantage to VSMCs in response to several proatherogenic stimuli. Additionally, we found that IRA might associate with IGF-IR favoring atherogenic actions of IGF-II [21]. However, the specific role of each IR isoform in VSMC migration remains unknown. We therefore analyzed, in the current study, the effect of insulin or IGFs on the migration of murine aortic VSMC lines, as well as the contribution to this process of IR isoforms, IGF-IR and hybrid receptors (IRA/IGF-IR and IRB/IGF-IR). Subsequently, we wondered whether some of the mechanisms studied in vitro could be of any in vivo relevance. For this purpose, we firstly analyzed IRA, IRB and IGF-IR mRNA expression by qRT-PCR in aorta from ApoE^−/−^ mice at 8, 12, 18 and 24 weeks of age. Then, we studied comparatively IR, IGF-IR and α-smooth muscle actin (α-SMA) expression in aortic roots from 8- and 24-week-old ApoE^−/−^ mice. Finally, we analyzed the mRNA expression of total IR, specifically IRB isoform, IGF-IR and IGFs in the medial layer of human aortic segments. Our results strongly suggest that the overexpression of IGF-IR or IRA isoform, as homodimers or as part of IRA/IGF-IR hybrid receptors, confers a migratory advantage to VSMCs that might be of relevance in early stages of atherosclerotic process.

## Methods

### Tissue samples

Twenty-eight human infradiaphragmatic aortic segments (medial layers of 8 healthy, 9 fatty streaks and 11 fibrolipidic lesions) each harvested from a different donor after organ transplantation with the authorization of the French Biomedicine Agency (authorization number PFS09-007), were included in this study. There were no significant differences in terms of age and gender. The investigation conforms to the principles outlined in the Declaration of Helsinki.

### Experimental model

Male mice were maintained in the Animal Care Facility under the standard conditions of temperature and 12 h light/dark cycle. All animals used are under C57BL/6 genetic background. Six week-old male ApoE^−/−^ knockout mice and their control (C57BL/6 mice) were fed a Western type diet (A04+ 21% kcal from fat) for 2, 6, 12 or 18 weeks respectively. All animal experimentation described in this manuscript was conducted according with accepted standards of human animal care, as approved by the corresponding institutional committee. The investigation also conforms to the Guide for the Care and Use of Laboratory Animals published by the National Institutes of Health (NIH Publication No. 85–23, revised 1996) and in accordance with The ARRIVE Guideline for Reporting Animal research.

Plasma levels of insulin were analyzed using ELISA kits (Millipore). Cholesterol and triglycerides were tested in plasma samples from fasted mice (Spinreact, Barcelona, Spain). Blood glucose level was determined in fasted animals using an automatic monitor (Roche Molecular Biochemicals GmbH, Mannheim, Germany).

### Cell cultures

Generation of immortalized IRLoxP^+/+^, IR^−/−^, IRA and IRB VSMC lines was previously described [17]. Briefly, primary VSMCs were obtained from thoracic aorta arteries of 3 male 8-week-old IRLoxP^+/+^ mice. Anesthetized mice (Avertin, 250 mg/kg, ip) were saline perfused and thoracic aorta arteries were submitted to collagenase dispersion and primary culture. Then, primary culture of IRLoxP^+/+^ VSMCs were immortalized by transfection with pBabe retroviral vector encoding SV40 Large T antigen and selected with 1 μg/mL puromycin for 3 weeks. Immortalized IRLoxP^+/+^ VSMCs were infected with adenoviruses encoding Cre recombinase to obtain IR^−/−^ VSMCs. Finally, IR^−/−^ VSMCs were transfected with pBABE retroviral vector encoding the individual spliced isoforms of the human IR, IRA or IRB, and selected with 200 μg/mL hygromycin for 2 weeks to obtain IRA or IRB VSMCs respectively.

Four cell lines were cultured to subconfluence (70–80%) with 10% (fetal bovine serum) FBS-DMEM for signaling studies and were serum and glucose starved for 4–5 h and then stimulated with insulin (10 or 100 nmol/L, Sigma-Aldrich Corp.), IGF-I (10 nmol/L, Millipore) or IGF-II (10 nmol/L, Millipore).

### Western blot analysis

Western blot analyses were performed on protein extracts from VSMCs as previously described [17]. The antibodies used were anti-IRβ and anti-IGF-IRβ from Santa Cruz Biotechnology (Dallas, TX, USA); anti-β-actin from Sigma-Aldrich Corp. (St. Louis, MO); anti-phospho-Akt (Thr308), anti-phospho-p70 S6 Kinase (Ser389) and anti-phospho-p44/42 MAPK (Thr202/Tyr204) from Cell Signaling (Danvers, MA, USA).

### Wound healing assays

Cells were cultured to confluence in 10% FBS-DMEM and serum deprived for 18 h. Then, the confluent monolayer was scratched with a sterile pipette tip and cells were washed twice with PBS and fresh medium was added. Cells were stimulated with insulin (10 nmol/L), IGF-I (10 nmol/L) or IGF-II (10 nmol/L) and maintained for 24 h at 37 °C and 5% CO_2_. Migration was followed by phase-contrast microscopy (Eclipse TE300 Nikon microscope coupled to a digital sight DS-U2 camera) at different time points (0, 6, 12 and 24 h) up to wound healing closure. Photographs were taken to quantify (using TScratch program) the percentage of wound healing closure at the different time points in relation to time 0.

### RNA extraction and real-time quantitative polymerase chain reaction

Total RNA was isolated from tissue samples using TRIzol reagent (Invitrogen, Carlsbad, CA, USA) and quantified by absorbance at 260 nm. One microgram of RNA was used to perform the reverse transcription with a High Capacity cDNA Archive kit (Applied Biosystems, Foster City, CA, USA). Real-time quantitative PCR (qRT-PCR) was performed on an ABI Prism 7500 sequence detection PCR system (Applied Biosystems) according to the manufacturer’s protocol, using the ΔΔCt method as previously described [17]. Quantification of IGF-IR, and IRA and IRB isoform mRNA levels in aorta from experimental model were performed by amplification of cDNA with TaqMan probes. The quantification of human IR, IRB isoform, IGF-IR, IGF-I and IGF-II mRNA levels was performed by amplification of cDNA using SYBR^®^ Premix Ex Taq™ (Takara Bio Inc., Otsu, Japan). The mRNA levels of target genes were normalized to 18S mRNA content. Thus, the amount of target, normalized to 18S and relative to the control, is given by real-time quantitative (RQ) = 2^−ΔΔCt^; ΔCt (cycle threshold) = Ct (target gene) − Ct (18S); ΔΔCt = ΔCt for any sample − ΔCt for the control. Sequences of primers are the following: IR: forward primer: 5´-CGAGAAGACCATCGACTCGG-3´ and reverse primer 5´-GACACCAGAGCGTAGGATCG-3´; IRB: forward primer: 5´-GAGGATTACCTGCACAACGTG-3´ and reverse primer 5´-TAGGGTCCTCGGCACCAG-3´; IGF-IR: forward primer: 5´-ATGCGGTGTCCAATAACTAC-3´ and reverse primer 5´-TTGTTGATGGTGGTCTTCTC-3´; IGF-I: forward primer: 5´-TTTCAACAAGCCCACAGGGT-3´ and reverse primer 5´-TTGAGGGGTGCGCAATACAT-3´;IGF-II: forward primer: 5´-GTCATGGCAGACGCCACATT-3´ and reverse primer 5´-CGAAGGCTCTGCCCTTCTTA-3´; and Housekeeping gene as 18S: forward primer: 5´-CCGTCGTAGTTCCGACCATAA-3´ and reverse primer 5´-CAGCTTTGCAACCATACTCCC-3´.

### Histological analysis

Aortic roots were OCT-embedded and sections of 7 μm interval were Oil-Red-O/hematoxylin stained to measure lipid depot. The lesion size on aortic root was measured as described [21]. IR and IGF-IR were detected by immunoperoxidase with rabbit anti-IRβ (sc-711) and anti-IGF-IRβ (sc-713) polyclonal antibodies. We also measured α-SMA with Anti-Actin, α-Smooth Muscle-Cy3™ antibody, mouse monoclonal (C6198, Sigma-Aldrich) to localize smooth muscle cells in aortic roots and check whether such cells had migrated from media to intima. To localize smooth muscle cells with positive staining for IR or IGF-IR, we performed immunofluorescence against IR or IGF-IR using anti-rabbit FITC (green staining) followed by immunofluorescence against Anti-Actin, α-Smooth Muscle-Cy3™ antibody (red staining) and finally, incubation with DAPI to stain nuclei (blue staining).

### Gelatin zymography

To determine MMP-2 and MMP-9 activities, 80% confluent cells were serum-deprived for 12–24 h and the culture medium of different experimental conditions was used for an electrophoresis in 10% SDS–polyacrylamide gels polymerized in the presence of 0.1% gelatin (Sigma-Aldrich) under non-reducing conditions. Gels were washed with 2.5% Triton X-100 for 30 min to remove SDS, rinsed with substrate buffer (0.2 M NaCl, 5 mM CaCl_2_, 1% Triton X-100, 0.02% NaN_3_, 50 mM Tris pH 7.5) and incubated in this buffer at 37 °C overnight to allow protein renaturation and MMP activation. To visualize gelatin degradation, gels were stained with Coomassie Brilliant Blue R-250 (Bio-Rad) for 60 min. MMP-2 and MMP-9 activities were quantified by Image J Program.

### Statistical analysis

All values are expressed as mean ± SEM. Differences between two groups were assessed using unpaired two-tailed *t*-tests. Data involving more than two groups were analyzed using a one-way ANOVA followed by a Bonferroni test if differences were noted (GraphPad Prism). The null hypothesis was rejected when p < 0.05.

## Results

### Differential insulin and IGFs signaling by IRA or IRB isoforms in VSMCs

To study the role of IR isoforms in the migration of vascular smooth muscle cells four murine aortic VSMC lines were used: bearing IR (IRLoxP^+/+^ VSMCs), lacking IR (IR^−/−^ VSMCs), specifically expressing IRA isoform (IRA VSMCs) or alternatively expressing IRB isoform (IRB VSMCs). Western blot analysis of IRβ confirmed deletion in IR^−/−^ VSMCs and expression of IR in reconstituted IRA and IRB VSMC lines (Additional file 1: Figure S1A). Regarding IGF-IR, a significantly higher expression was found in IR^−/−^ and IRB VSMCs compared to IRLoxP^+/+^ VSMCs, while cells exclusively expressing IRA showed lower level of IGF-IR protein (Additional file 1: Figure S1A).

We studied the signaling pathways induced by insulin or IGFs. Cells were stimulated with 10 nmol/L insulin, IGF-I or IGF-II for 10 min and phosphorylation of Akt, p70S6 K and p44/42 MAPK was analyzed. As expected, IR^−/−^ VSMCs responded to IGFs, but did not to insulin, in regards to Akt activation. However, a significant increase of p-Akt, p-p70S6 K, and p-p44/42 MAPK was induced by the three stimuli in IRLoxP^+/+^ VSMCs (Additional file 1: Figure S1B). More importantly, IRA VSMCs showed a significantly higher activation of Akt, p70S6 K and p44/42 MAPK in response to insulin, IGF-I or IGF-II, than in IRB VSMCs (Additional file 1: Figure S1B).

### Differential contribution of IRA, IRB and IGF-IR to VSMC migration in response to insulin or IGFs

**Fig. 1:**
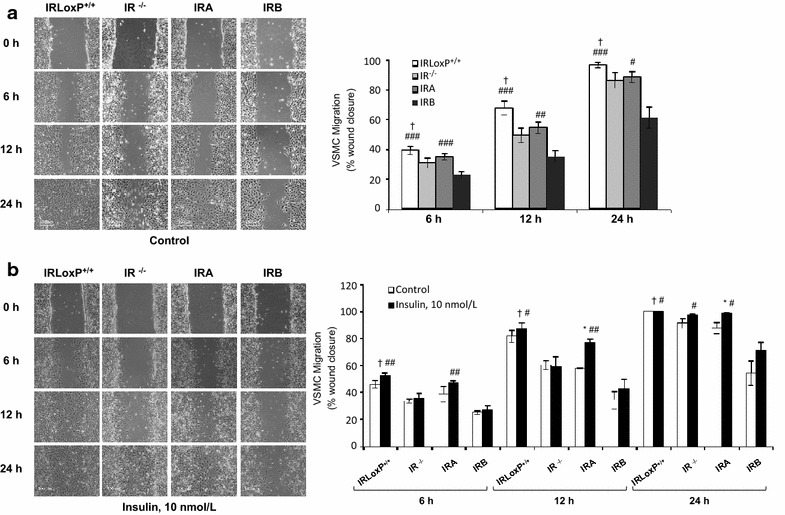
Insulin induces migration in IRA VSMCs. **a** Analysis of basal migration of four VSMC lines at 6, 12 and 24 h. The experiments were performed by wound healing assays and the percentage of wound healing closure quantified (using TScratch program) at different time points. Experiments were performed 4–7 times. **b** Effect of insulin on wound healing closure in four VSMC lines at 6, 12 and 24 h. Photomicrographs (10x magnification) were selected the more representative. Experiments were performed at least 3 times. *p < 0.05 vs. each control; ^†^p < 0.05 vs. IR^−/−^ VSMCs; ^#^p < 0.05, ^##^p < 0.005, ^###^p < 0.001 vs. IRB VSMCs

**Fig. 2:**
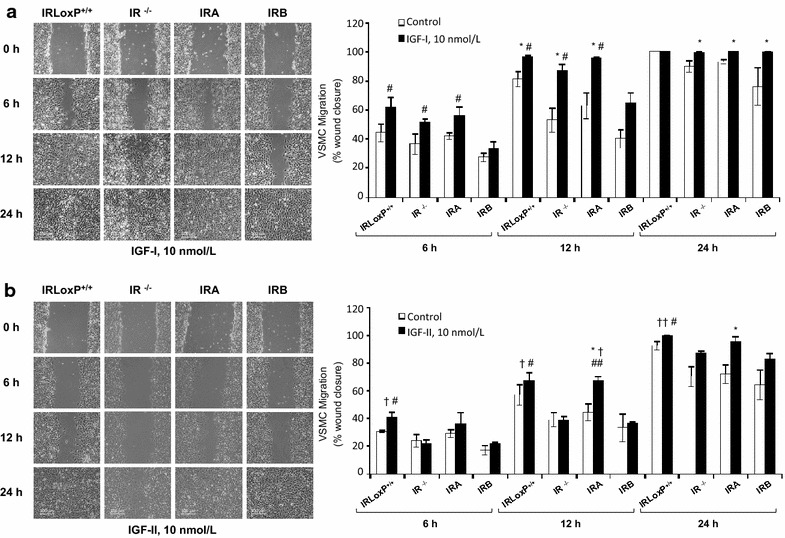
IGF-I strongly induces VSMC migration and IGF-II has a lesser effect on IRA VSMC migration. Effect of IGF-I (**a**) or IGF-II (**b**) on wound healing closure in four lines of VSMCs at 6, 12 and 24 h. Photomicrographs (10x magnification) were selected the more representative. Experiments were performed at least 3 times. *p < 0.05 vs. each control; ^†^p < 0.05, ^††^p < 0.005 vs. IR^−/−^ VSMCs; ^#^p < 0.05, ^##^p < 0.005 vs. IRB VSMCs

Proliferation and migration of VSMCs contribute to atherosclerotic process. In this regard, we previously demonstrated that IRA isoform confers a proliferative advantage to those cells [17]. Then, we hypothesized that a differential role of IR isoforms might also occur in the migration of VSMCs. To address this issue, we measured the migration rate of four VSMC cell lines at 6, 12 and 24 h by wound healing assays. Maximal migration was observed in IRLoxP^+/+^ VSMCs as compared with IR^−/−^ and IRB VSMCs. IRA VSMCs showed a significantly higher migration than IRB VSMCs (Fig. 1a). Moreover, we assessed the effect of insulin or IGFs stimulation on VSMC migration. Firstly, we found a significant increase of insulin-induced migration in IRA VSMCs at 12 and 24 h after wounding. IRLoxP^+/+^ and IRB VSMCs exhibited a slightly response to insulin and no effect was observed in IR^−/−^ VSMCs (Fig. 1b). IGF-I strongly induced migration of all cell lines studied with significant increases in the percentage of wound closure at 12 and 24 h (Fig. 2a). Given the fact that IGF-I enhanced migration of cells lacking IR, the effect exerted by IGF-I might be mediated through IGF-IR. In contrast, IGF-II showed a discrete effect on VSMC migration. However, IGF-II induced IRA VSMC migration significantly faster than IRB VSMCs at 12 h (Fig. 2b). These results suggest a differential contribution of IR and IGF-IR to VSMC migration, the latter being the main mediator of IGF-I-induced migration. Conversely, insulin and IGF-II seem to require IR, and more specifically IRA isoform, to induce migration of VSMCs.

One of the mechanisms that may also partially explain the differential migration between IRA and IRB VSMCs is a differential activation of certain matrix metalloproteinases (MMPs), specifically the gelatinases such as MMP-2 and MMP-9, which have been involved in VSMC migration [22–24]. Thus, we observed by gelatin zymography that MMP-2 activity was much higher in IRA than IRB VSMCs under basal conditions or upon stimulation with insulin, IGF-I or IGF-II for 12 and 24 h (Additional file 2: Figure S2).

### Inhibition of IGF-IR strongly reduces VSMC migration

**Fig. 3:**
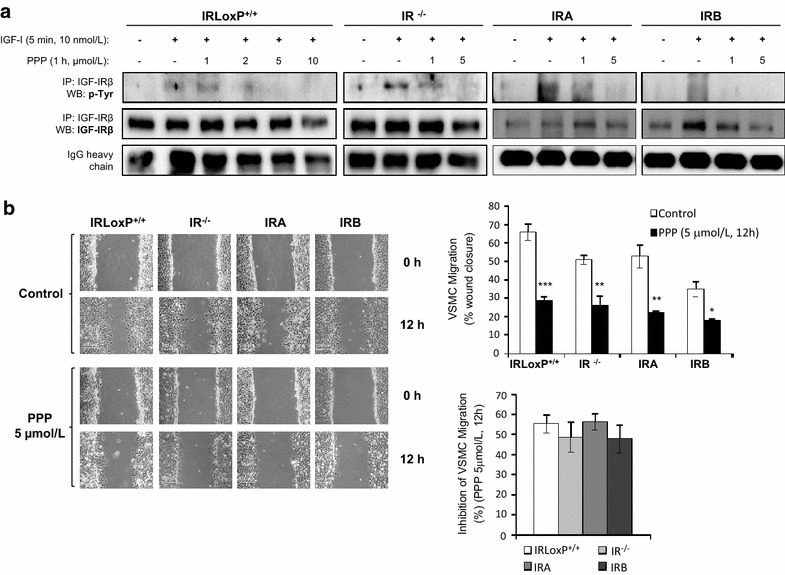
IGF-IR contributes to basal VSMC migration. **a** Effect of PPP (IGF-IR inhibitor) at different doses on IGF-IR tyrosine phosphorylation of four VSMC lines. We performed the immunoprecipitation against IGF-IRβ followed by Western blot against p-tyrosine. **b** Effect of PPP on basal VSMC migration at 12 h by wound healing closure and the percentage of inhibition of VSMC migration induced by PPP. Experiments were performed 4–7 times. *p < 0.05, **p < 0.005, ***p < 0.001 vs. each control

To further investigate the role of IGF-IR in VSMC migration, we utilized a highly specific inhibitor of IGF-IR tyrosine phosphorylation, picropodophyllin (PPP), without affecting IR. A PPP dose–response curve revealed a complete inhibition of IGF-IR phosphorylation at 5 and 10 µmol/L in IRLoxP^+/+^ VSMCs upon stimulation with IGF-I. Similarly, 5 µmol/L PPP completely avoided IGF-IR tyrosine phosphorylation in all others cell lines (Fig. 3a). Therefore, cells were treated with 5 µmol/L PPP for 12 h to address migration experiments. We found a notable and significant decrease of VSMC migration induced by PPP treatment, reaching roughly 50% of inhibition in all the four cell lines migration (Fig. 3b). These data indicate that IGF-IR mostly contributes to VSMC migration under basal conditions.

**Fig. 4:**
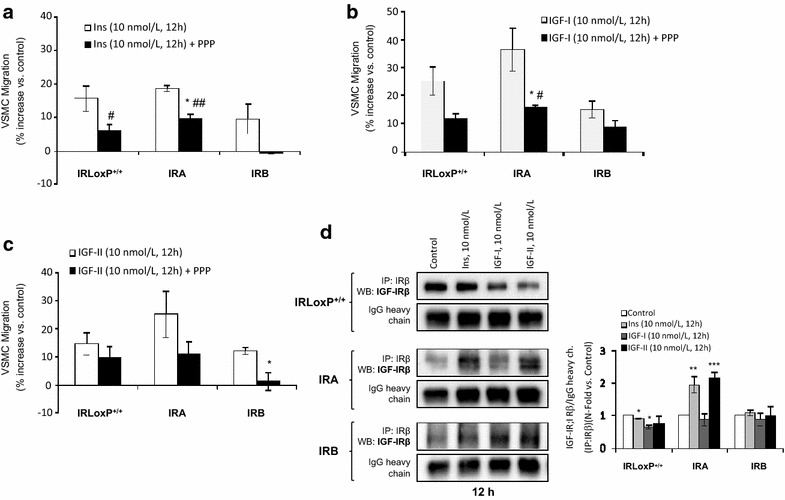
Effect of IGF-IR inhibition on VSMC migration induced by insulin or IGFs. Quantification of PPP effect on VSMC migration stimulated with insulin (**a**), IGF-I (**b**) or IGF-II (**c**) at 12 h by wound healing closure. *p < 0.05 vs. each control; ^#^p < 0.05, ^##^p < 0.005 vs. IRB VSMCs. **d** Formation of hybrid receptors (IRA/IGF-IR or IRB/IGF-IR) in IRLoxP^+/+^, IRA and IRB VSMCs stimulated with insulin, IGF-I or IGF-II. We performed the immunoprecipitation against IRβ followed by Western blot against IGF-IRβ. Experiments were performed at least 3 times. *p < 0.05, **p < 0.005, ***p < 0.001 vs. each control

A step further, we studied the effect of IGF-IR inhibition on the VSMC migration stimulated by insulin or IGFs. Interestingly, PPP treatment completely abolished insulin-induced migration in IRB VSMCs, whereas migration of IRLoxP^+/+^ or IRA VSMCs was partially inhibited (Fig. 4a), suggesting that IRB, but not IRA, requires the presence of IGF-IR to induce migration in response to insulin. Regarding IGF-I-mediated migration, a reduction of approximately one half was the result of IGF-IR inhibition in all cell lines analyzed and no significant differences were noted between IRA and IRB VSMCs (Fig. 4b). Finally, PPP caused a much higher inhibition of IGF-II-induced migration in IRB VSMCs than that observed in IRLoxP^+/+^ or IRA VSMCs (Fig. 4c).

### Insulin and IGF-II induces the formation of IRA/IGF-IR hybrid receptors in VSMCs

To address the issue of hybrid receptor formation, lysates from IRLoxP^+/+^, or IRA, or IRB VSMCs, were immunoprecipitated with anti-IRβ antibody followed by Western blot against IGF-IR. All cell lines showed a basal association between IR and IGF-IR. Nevertheless, expression of IRA/IGF-IR hybrid receptors was significantly increased only in IRA VSMCs upon stimulation with insulin or IGF-II, but not with IGF-I, for 12 h (Fig. 4d). In addition, expression of both IR and IGF-IR was measured in total protein extracts and detected an increase of IRA isoform, with an equal expression of IGF-IR, in response to insulin or IGF-II (Additional file 3: Figure S3). These findings are consistent with the greater migration observed in IRA VSMCs upon stimulation with insulin or IGF-II for 12 h (Figs. 1b, 2b).

### Role of IR isoforms and IGF-IR in VSMC migration in an experimental model of atherosclerosis

To assess the role of IR isoforms and IGF-IR in experimental atherosclerosis, we used ApoE^−/−^ mice at 8, 12, 18 and 24 weeks of age. This model showed hypercholesterolemia and hypertriglyceridemia at all weeks of age without alterations in body weight, glucose or insulin plasma levels (see Table 1).

**Table 1:** Characterization of an experimental model of atherosclerosis

	8 weeks		12 weeks		18 weeks		24 weeks	
	Control (n = 5)	ApoE^−/−^ (n = 5)	Control (n = 8)	ApoE^−/−^ (n = 8)	Control (n = 8)	ApoE^−/−^ (n = 8)	Control (n = 8)	ApoE^−/−^ (n = 8)
Body weight (g)	20.4 ± 0.1	21.3 ± 0.6	24.7 ± 0.5	24.3 ± 0.5	27.7 ± 1	26.5 ± 0.3	28.3 ± 1	30.5 ± 1
BAT (mg)/BW (g)	3.9 ± 0.02	2.8 ± 0.6	4.3 ± 0.4	3.7 ± 0.4	4.1 ± 0.7	3.0 ± 0.1	4.7 ± 0.5	3.4 ± 0.5
WAT (mg)/BW (g)	10.5 ± 0.8	11 ± 0.6	12.2 ± 2.7	17 ± 0.2	14.7 ± 2	17.5 ± 1	22 ± 4	27 ± 1
Glucose (mg/dL)	80.5 ± 6.5	79 ± 13	83 ± 4	104 ± 15	110.9 ± 12	106 ± 4	117 ± 1	101 ± 11
Insulin (ng/mL)	0.24 ± 0.003	0.3 ± 0.1	0.25 ± 0.02	0.32 ± 0.02	0.26 ± 0.02	0.35 ± 0.04	0.24 ± 0.02	0.3 ± 0.01
Cholesterol (mg/dL)	79.1 ± 13	461 ± 39*	109.4 ± 8.4*	565.3 ± 48.8*	122.05 ± 12*	569.8 ± 18*	120 ± 5*	603.9 ± 31**
TG (mg/dL)	43.4 ± 1.6	53.9 ± 25*	36.6 ± 5	56.9 ± 8*	62.3 ± 12	73.2 ± 5*	57.2 ± 8	110 ± 12**

Male ApoE^−/−^ mice were fed with Western type diet since 6 week of age for 2 weeks (control and ApoE^−/−^ at 8 weeks of age), 6 weeks (control and ApoE^−/−^ at 12 weeks of age), 12 weeks (control and ApoE^−/−^ at 18 weeks of age) or 18 weeks (control and ApoE^−/−^ at 24 weeks of age). We measured the body weight, BAT (mg)/BW (g), WAT (mg)/BW (g), glucose, insulin, cholesterol and triglycerides plasma levels the same day of sacrifice in fasted mice. ApoE^−/−^ mice at all weeks of age showed hypercholesterolemia and hypertriglyceridemia and the other parameters with no significant differences in relation to each their controls

*BW* body weight, *BAT* brown adipose tissue, *WAT* white adipose tissue, *TG* triglycerides

* p < 0.05, ** p < 0.005 vs. each control

**Fig. 5:**
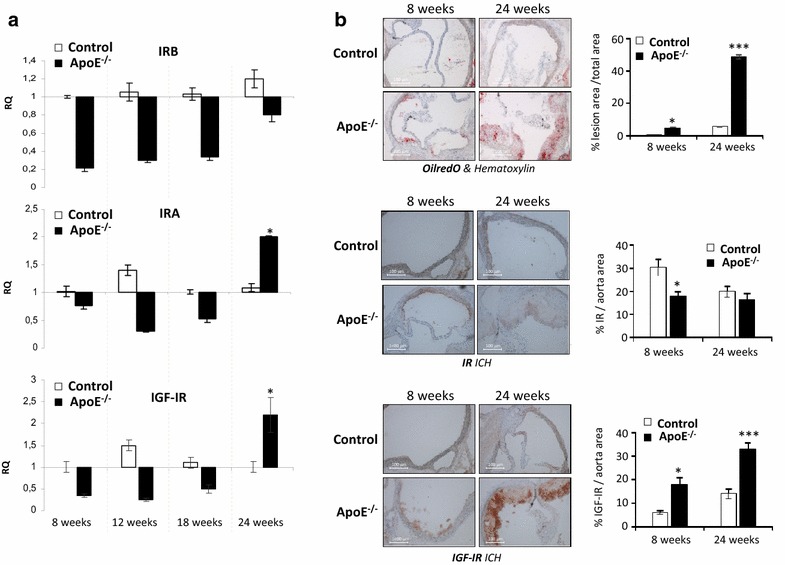
IR and IGF-IR expression in aorta from an experimental model of atherosclerosis. **a** By qRT-PCR, we studied IRA, IRB and IGF-IR mRNA expression in aorta from ApoE^−/−^ mice at 8, 12, 18 and 24 weeks of age and their respective controls. *p < 0.05 vs. each control. **b** Representative photomicrographs (10× magnification) and quantifications of OilRedO staining and immunohistochemistry of IR and IGF-IR in aortic roots from ApoE^−/−^ at 8 and 24 weeks of age and their respective controls. *p < 0.05 vs. each control; ***p < 0.001 vs. each control. *AE* ApoE^−/−^ mice,* C* control mice. C 8ws (n = 5); AE 8ws (n = 5); C12 ws (n = 5); AE12 ws (n = 8); C18 ws (n = 8); AE 18ws (n = 8); C 24ws (n = 8); AE 24ws (n = 8)

Firstly, we studied the mRNA expression of IR isoforms and IGF-IR by qRT-PCR in aorta. A significant increase of IRA and IGF-IR mRNA was noted in 24-week-old ApoE^−/−^ mice. However, IRB was diminished in aorta from ApoE^−/−^ mice at all weeks of age (Fig. 5a). After that, we studied the lesion area and lipid deposits together with IR and IGF-IR protein levels only in aortic roots from 8- and 24-week-old ApoE^−/−^ mice and their respective controls, because it was previously described that at 15–20 weeks of age, ApoE^−/−^ mice might have fibrous plaques in which the presence of a fibrous cap containing VSMCs would indicate their migration from the media [25]. A progression of atherosclerosis was observed by OilRedO staining as a progressive increase of lesion area, stenosis and lipid deposits in aortic roots from 24-week-old ApoE^−/−^ vs. 8-week-old ApoE^−/−^ mice (Fig. 5b). By immunohistochemistry against total IR, we found a significant decrease of IR in aortic roots from 8-week-old ApoE^−/−^ mice (Fig. 5b), consistent with the decreased mRNA expression of both IRA and IRB isoforms in aorta from ApoE^−/−^ mice at 8 weeks of age (Fig. 5a). In aortic roots from 24-week-old ApoE^−/−^ mice, protein levels of total IR were similar to their controls (Fig. 5b), and this can be correlated with the increased expression of IRA at mRNA level, together with the decreased expression of IRB mRNA (Fig. 5a). In addition to the increased IGF-IR mRNA levels, a significant increase of IGF-IR protein was observed in aortic roots from ApoE^−/−^ mice at 24 weeks of age (Fig. 5a, b). However, besides VSMCs, many inflammatory cells can be present in those plaques and they might also express IR or IGF-IR.

**Fig. 6:**
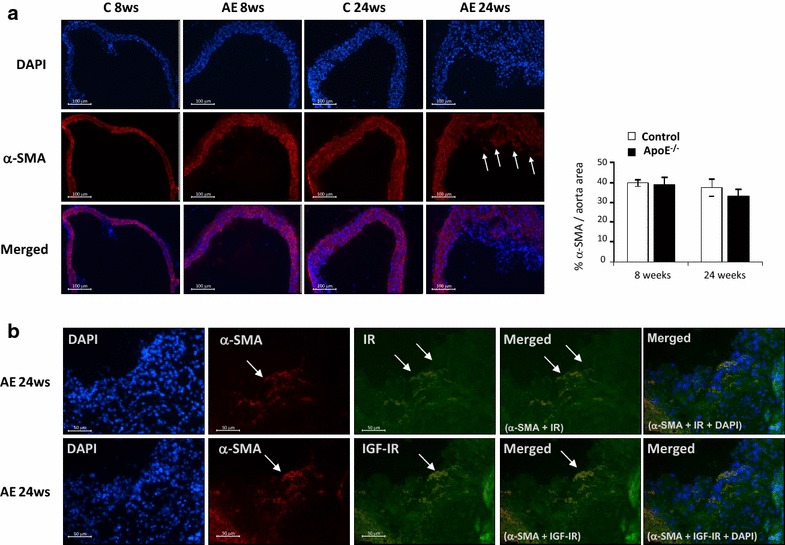
VSMCs migrated into the intima of atherosclerotic plaques express IR or IGF-IR. **a** Representative photomicrographs (10× magnification) and quantifications of immunofluorescence of α-SMA in aortic roots from ApoE^−/−^ mice at 8 and 24 weeks of age and their respective controls. **b** Representative photomicrographs (20× magnification) of double immunofluorescence against IR or IGF-IR (*green staining*) and α-SMA (*red staining*) in aortic roots from ApoE^−/−^ mice at 24 weeks of age. DAPI staining was performed to localize nuclei of cells presented in aortic roots (*blue staining*). *White arrows* indicate VSMCs in the intima of fibrous plaque from 24-week-old ApoE^−/−^ mice. *AE* ApoE^−/−^ mice, *C* control mice. C 8ws (n = 5); AE 8ws (n = 5); AE 18ws (n = 8); C 24ws (n = 8); AE 24ws (n = 8)

In order to localize VSMCs in aortic roots, we checked by immunofluorescence the expression of α-SMA, a well-known marker of smooth muscle cells. Very similar levels of α-SMA were found in aortic roots from ApoE^−/−^ mice at 8 and 24 weeks of age in relation to their respective controls (Fig. 6a). We also observed medial VSMCs migrated into the intima of fibrous plaques from 24-week-old ApoE^−/−^ mice (see white arrows of Fig. 6a). Moreover, by double immunofluorescence against IR or IGF-IR and α-SMA (Fig. 6b), we found that those VSMCs present in fibrous plaques from 24-week-old ApoE^−/−^ mice expressed IR or IGF-IR (see arrows and merged of Fig. 6b).

### mRNA expression of IGF-IR and IGFs in human aortic medial layers

Based in our in vitro and in vivo results and those previously published in ApoE^−/−^ or BATIRKO mice, two models showing vascular damage [21], we hypothesized that IR and its isoforms and IGF-IR could play a differential role in human early atherosclerosis. For this purpose, we analyzed the mRNA expression of total IR, specifically IRB isoform, IGF-IR and IGFs by qRT-PCR in the medial layer of human aortas (Additional file 4: Figure S4). Among 28 aortic samples, 8 were devoid of grossly visible signs of lesions and were classified as healthy, whereas all other samples showed macroscopic features of early atheromatous lesions, including 9 fatty streaks and 11 fibrolipidic lesions. We found that total IR mRNA expression was significantly lower in media from fibrolipidic lesion-bearing aortas as compared to healthy aortas. However, no significant changes were observed when we analyzed specifically IRB mRNA level. More importantly, a marked and significant increase in gene expression of IGF-I and IGF-IR was observed in medial layer from aortas bearing fatty streaks when compared with healthy aortas and fibrolipidic lesions. Furthermore, IGF-II mRNA expression was notably higher in fatty streaks than in healthy aortas and fibrolipidic lesions. These results strongly suggest that increased expression of IGF-IR and IGFs in tunica media, mainly composed of VSMCs, might contribute to migration of those cells into the intima.

## Discussion

Vascular injury results in changes in the vascular smooth muscle cell environment, including increases in growth factor availability and cell alterations, leading to proliferation and migration of VSMCs and thereby to an organized atherosclerotic plaque [26]. Consequently, elucidating the underlying regulators of these processes could provide a novel therapeutic strategy targeting VSMCs to prevent atherosclerotic progression and its clinical consequences. In the present paper, we addressed the issue of a better understanding of molecular mechanisms involved in VSMC migration, specifically the part played by IR isoforms and IGF-IR in this process. We used four aortic VSMC lines: IRLoxP^+/+^ VSMCs (bearing IR), IR^−/−^ VSMCs (lacking IR), IRA VSMCs (expressing IRA isoform) and IRB VSMCs (expressing IRB isoform) and we firstly studied the activation of PI3 K/Akt or MAPK, the major signaling pathways by which insulin and IGFs exert their effects on cellular metabolism, growth and survival [27]. We previously described that insulin induced a more sustained signaling in IRA compared with IRB neonatal pancreatic beta cell lines [28] and a higher stimulation of Akt or MAPK pathways in IRA than IRB VSMCs [21]. Now, we show that IRA isoform in VSMCs mediates a greater activation of Akt, p70S6K and p44/42 MAPK than IRB in response not only to insulin, but also to IGFs. Furthermore, phosphorylation of those kinases was highly pronounced when IRA VSMCs were stimulated with IGFs, especially with IGF-II. In this regard, IRA has been reported to exhibit high affinity for insulin, intermediate affinity for IGF-II and low affinity for IGF-I, whereas IRB is a highly specific receptor for insulin [13]. Moreover, in mouse fibroblast expressing only IRA and not IGF-IR, despite producing a lower IRA autophosphorylation than insulin, IGF-II induced a higher p70S6K/Akt activation ratio compared with insulin. IGF-I elicited a similar signaling pattern than IGF-II, although it binds IRA with much lower affinity than IGF-II [29].

Type 2 diabetes and the metabolic syndrome are well known risk factors for atherosclerosis in part due to insulin resistance and/or hyperinsulinemia [30, 31]. Additionally, accelerated atherosclerosis in diabetic patients has been associated with the fact that diabetic VSMCs exhibit significantly increased rates of proliferation, adhesion and migration [32]. In this sense, several authors have examined the effect of insulin on VSMC migration, but the results are controversial. Yang and Kahn reported that physiological concentrations of insulin did not affect the migration of cultured rat VSMC, but increased it when cells were also stimulated with Ang II or inhibited it in the presence of NO [33]. Furthermore, in bovine aortic VSMCs 10 nmol/L insulin moderately stimulated migration mainly through the MAPK pathway, whereas insulin maintained VSMC quiescence and differentiation via PI3K-dependent signaling [34]. In others studies, a high dose of insulin (100 nmol/L) was found to induce migration and proliferation of both human and rat VSMCs [35, 36]. Nevertheless, this supraphysiological concentration of insulin can stimulate IGF-IR, which is known to mediate VSMC migration [4]. In our work, migration studies showed that insulin stimulation led to a higher increased migration in IRA than IRB VSMCs and had no effect on IR^−/−^ VSMCs, indicating that insulin exerts its effect on VSMC migration by binding IR, mainly IRA isoform. IGF-I, by contrast, strongly promoted the migration of VSMCs and this effect appears to be mediated mainly through IGF-IR, since a significant increase in migration was observed in cells lacking IR. Consistent with this, it is well established that IGF-I is a potent stimulator of VSMC proliferation and migration as a result of binding its own receptor [4, 37, 38]. IGF-II has also been found to induce migration in VSMCs through IGF-IR, but it was sixfold less potent than IGF-I [4]. Because IGF-II stimulated migration only in IRA VSMCs, our findings indicate that IGF-II requires the presence of IRA isoform, as homodimers or as part of IRA/IGF-IR hybrid receptors, to induce the migration of VSMCs.

Migration assays in the presence of PPP, a highly specific inhibitor of IGF-IR, demonstrated the implication of this receptor in VSMC migration. PPP specifically blocks phosphorylation of tyrosine 1136 in the IGF-IR activation loop, which is necessary for autophosphorylation of others tyrosine residues of the β subunit, but do not interfere with IR activity [39, 40]. We found that IGF-IR inhibition abolished insulin- and IGF-II-induced migration in IRB VSMCs, whereas migration of IRA VSMCs was partially inhibited. However, IGF-I-stimulated migration was inhibited by PPP at a similar level in both IRA and IRB cell lines. Taking into account that IR/IGF-IR hybrid receptor autophosphorylation occurs by an intramolecular transreaction in which both β subunits transfer phosphate to each other [41], it might be considered that signal transduction through hybrid receptors, like IGF-IR homodimers, is prevented with PPP treatment. Therefore, remaining migration when IGF-IR phosphorylation was inhibited appears to be mediated by IRA, but not by IRB homodimers, upon stimulation with insulin or IGF-II.

Other authors have described the presence of IR/IGF-IR hybrid receptors in human or rat VSMCs [42, 43], and we have previously demonstrated that both IRA/IGF-IR and IRB/IGF-IR hybrid receptors are expressed in our VSMC lines [21]. Insulin and IGFs are able to stimulate IRA/IGF-IR hybrid receptors, although insulin is less effective. Regarding IRB/IGF-IR activation, insulin and IGF-II are much less effective than IGF-I [44]. We currently describe that IRA expression and thereby IRA/IGF-IR hybrid receptor formation, are increased by insulin or IGF-II, and this might favor the migration of VSMCs.

Human atherosclerotic disease has been histologically classified by the AHA into eight types of lesions, from early lesions, including initial and fatty streak lesions, to advanced atherosclerotic lesions [45]. In early atherosclerotic lesions, IGF-IR might contribute to atherosclerotic progression by mediating proatherogenic actions of IGFs. Other studies have described an increased level of IGF-IR expression in VSMCs of atherosclerotic plaques of rabbit aortas [46], as we have observed in both aorta and aortic roots from ApoE^−/−^ mice at 24 weeks of age. Moreover, increased IGF-IR and IGF-I mRNA transcripts were observed in asymptomatic than in symptomatic plaque VSMCs [47]. In contrast, IGF-IR and IGF-I are decreased in advanced atherosclerotic plaques and this is consistent with the increased apoptotic rates of VSMCs, potentially leading to plaque weakening, plaque rupture and acute coronary events [48, 49]. In the same way, a recent study showed that a monocyte/macrophage specific IGF-IR knockout mice on ApoE^−/−^ background significantly increased atherosclerotic lesion formation and changed plaque composition to a less stable phenotype [50]. Regarding IGF-II, it has been described as a pivotal promoter of growth of the atherosclerotic lesion in ApoE^−/−^ mice and local overexpression of IGF-II can induce the appearance of aortic focal intimal masses [51]. In this sense, we describe that IRA isoform expression is significantly increased in aorta from 24-week-old ApoE^−/−^ mice, which it might favor proatherogenic actions of IGF-II. Consistently, we show for the first time that gene expression of IGF-I, IGF-II and IGF-IR is notably increased in the medial layer, mainly composed of VSMCs, of human aortas bearing early atherosclerotic lesions. Additionally, in ApoE^−/−^ mice we observed that VSMCs migrated into the intima of fibrous plaques showed positive staining for IR and IGF-IR. Overall, increased expression of IRA and IGF-IR would be required to support the migration of medial VSMCs into the intima.

## Conclusions

**Fig. 7:**
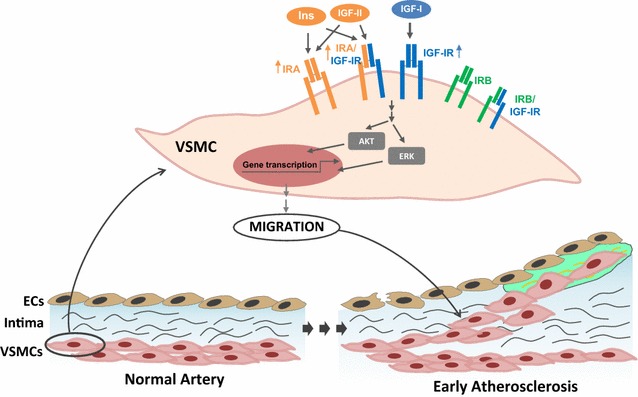
Proposed model of role of IR isoforms, IGF-IR and IR/IGF-IR hybrid receptors in VSMC migration. We propose that IGF-I mainly through IGF-IR, while insulin and IGF-II by binding IRA homodimers or IRA/IGF-IR hybrid receptors might favor VSMC migration and contribute to plaque growth in early states of atherosclerosis. *ECs* endothelial cells, *VSMCs* vascular smooth muscle cells

In conclusion, our in vitro results strongly suggest that IGF-I is a potent inducer of VSMC migration mainly through IGF-IR, and to a lesser extent through IR. Insulin and IGF-II are less potent and require the presence of IRA isoform to induce migration, by binding IRA homodimers or IRA/IGF-IR hybrid receptors. Therefore, overexpression of IGF-IR or IRA isoform might occur in early stages of atherosclerosis favoring the migration and proliferation of VSMCs and thereby atherosclerotic progression of relevance in experimental mouse models and in humans (Fig. 7). Whether IRA/IGF-IR hybrid receptors play a direct role at the early stage of atherogenesis in humans remains to be established.

## Additional files

**Additional file 1.** Characterization and insulin signaling in VSMC lines. (A) Western blot analysis of IR and IGF-IR in four VSMC lines. *p<0.05 vs IRLoxP^+/+^ VSMCs; †p<0.05, ††p<0.005 vs. IRA VSMCs. Immunoprecipitation of IRβ in IRLoxP^+/+^ or IR^-/-^ (B) Representative gels of Western blot analysis and its quantifications of Akt (T308), p70S6K (T389) and p44/42 MAPK (T202/Y204) phosphorylation in four cell lines stimulated with insulin, IGF-I or IGF-II. Experiments were performed at least 3 times. *p<0.05, **p<0.005, ***p<0.001 vs each control.

**Additional file 2.** Measure of MMP-2 and MMP-9 activities by gelatin zymography in IRA and IRB VSMCs stimulated with insulin, IGF-I or IGF-II for 12 or 24h. FBS was used as a positive control.

**Additional file 3.** Western blot analysis of IR and IGF-IR expression in IRLoxP^+/+^, IRA and IRB VSMCs stimulated with insulin, IGF-I or IGF-II for 12h.

**Additional file 4.** Increased expression of IGF-IR and IGFs in media from human aortas bearing fatty streaks. Analysis of mRNA expression of total IR, IRB isoform, IGF-IR and IGFs by qRT-PCR in the medial layer of 28 human aortas (8 healthy, 9 fatty streaks and 11 fibrolipidic lesions). *p<0.05 vs. healthy aorta; †p<0.05 vs. fatty streak.

## Abbreviations

α-SMA: α-smooth muscle actin
Akt: protein kinase B
ApoE^−/−^: apolipoprotein E knockout
BATIRKO: brown adipose tissue-specific insulin receptor knockout
IGF-IR: insulin-like growth factor-I receptor
IGFs: insulin-like growth factors
IR: insulin receptor
MMPs: matrix metalloproteinases
qRT-PCR: real-time quantitative PCR
VSMCs: vascular smooth muscle cells
