# Supplementary figures and images for: Expression of insulin receptor (IR) A and B isoforms, IGF-IR, and IR/IGF-IR hybrid receptors in vascular smooth muscle cells and their role in cell migration in atherosclerosis
Source: Cardiovasc Diabetol. 2016 Dec 1;15:161. doi: 10.1186/s12933-016-0477-3 (PMC5134076; doi:10.1186/s12933-016-0477-3)

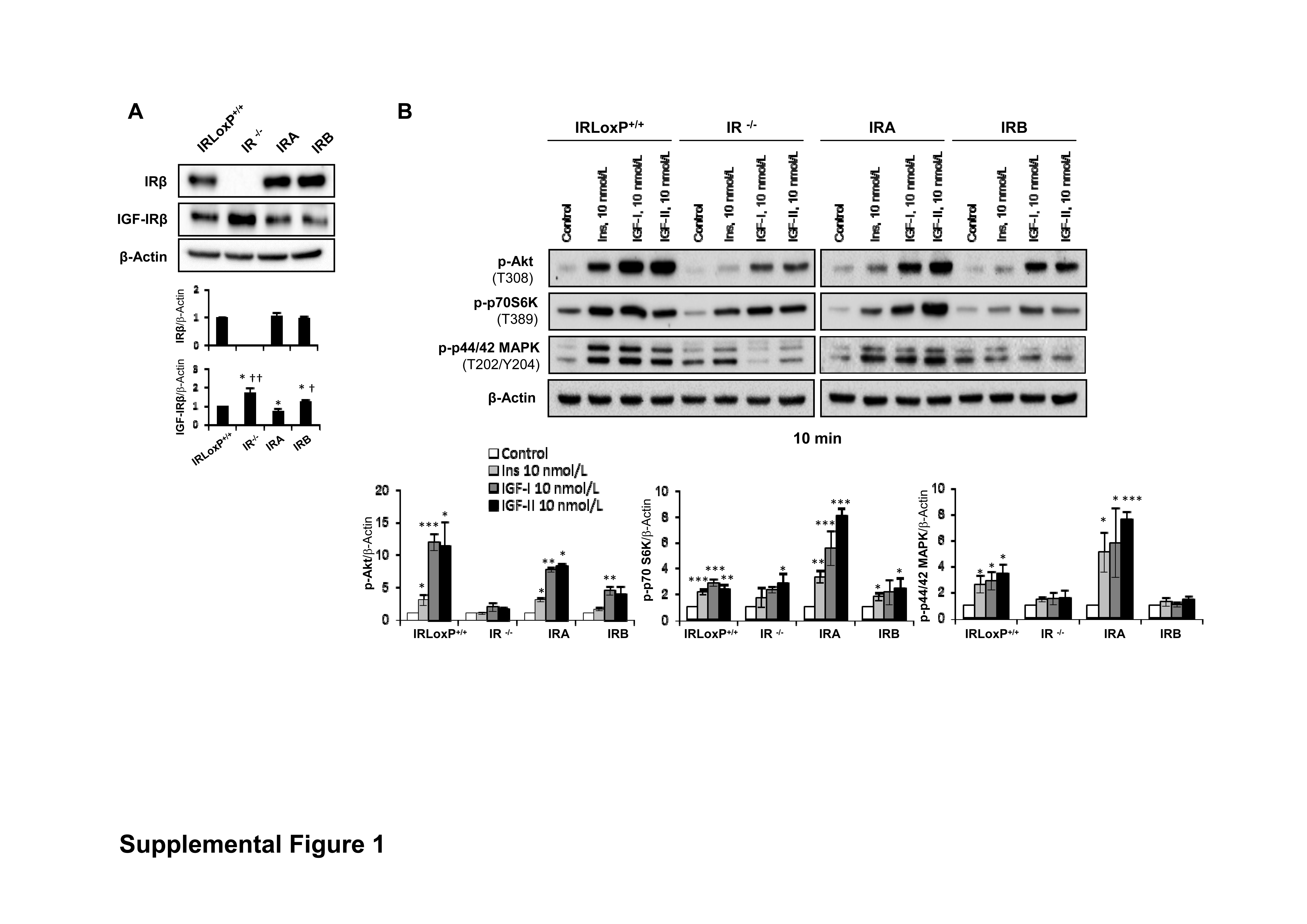

Supplement: Supplementary file 1 — Additional file 1. Characterization and insulin signaling in VSMC lines. (A) Western blot analysis of IR and IGF-IR in four VSMC lines. *p<0.05 vs IRLoxP+/+ VSMCs; †p<0.05, ††p<0.005 vs. IRA VSMCs. Immunoprecipitation of IRβ in IRLoxP+/+ or IR-/- (B) Representative gels of Western blot analysis and its quantifications of Akt (T308), p70S6K (T389) and p44/42 MAPK (T202/Y204) phosphorylation in four cell lines stimulated with insulin, IGF-I or IGF-II. Experiments were performed at least 3 times. *p<0.05, **p<0.005, ***p<0.001 vs each control. [file 12933_2016_477_MOESM1_ESM.tif]

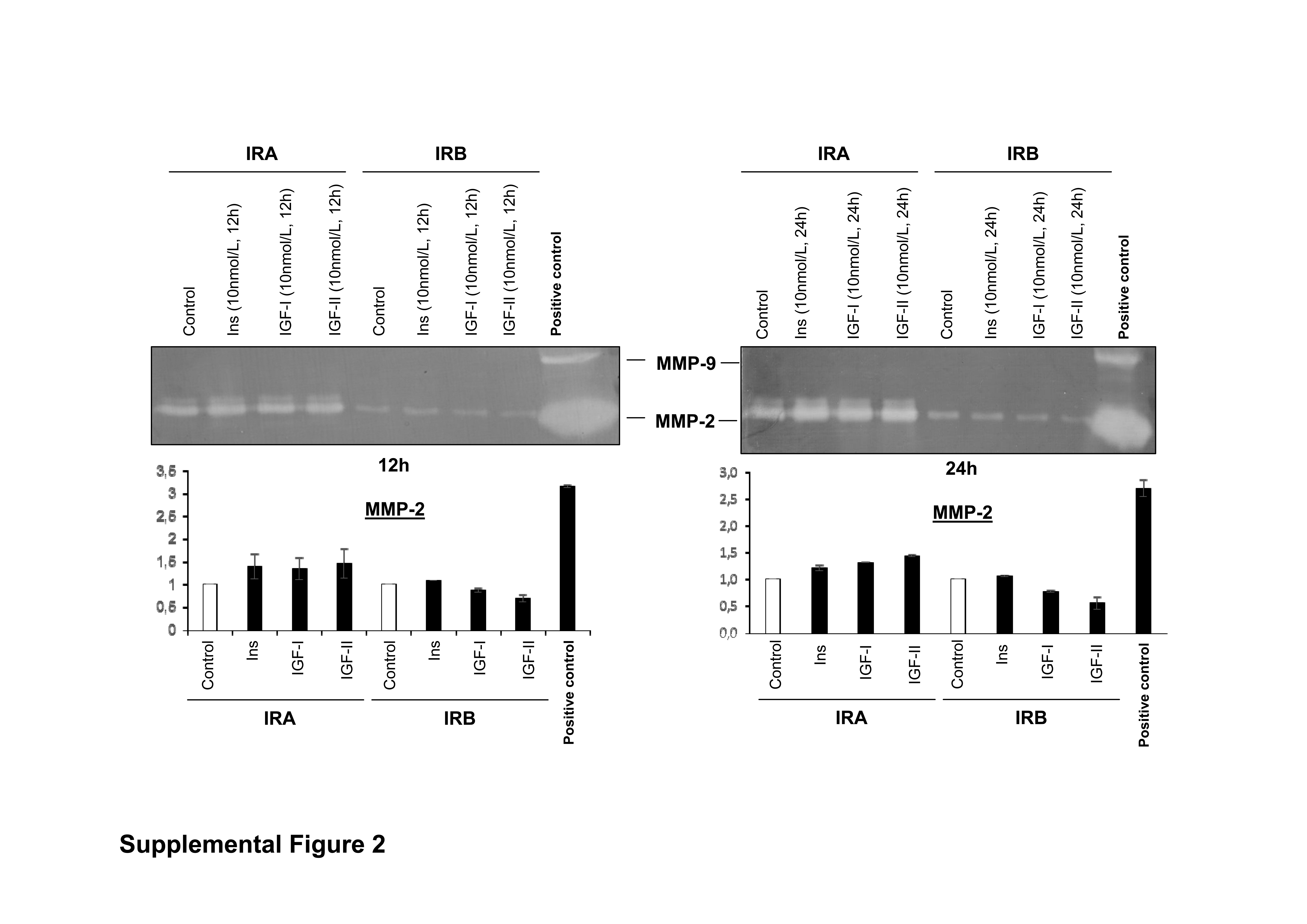

Supplement: Supplementary file 2 — Additional file 2. Measure of MMP-2 and MMP-9 activities by gelatin zymography in IRA and IRB VSMCs stimulated with insulin, IGF-I or IGF-II for 12 or 24h. FBS was used as a positive control. [file 12933_2016_477_MOESM2_ESM.tif]

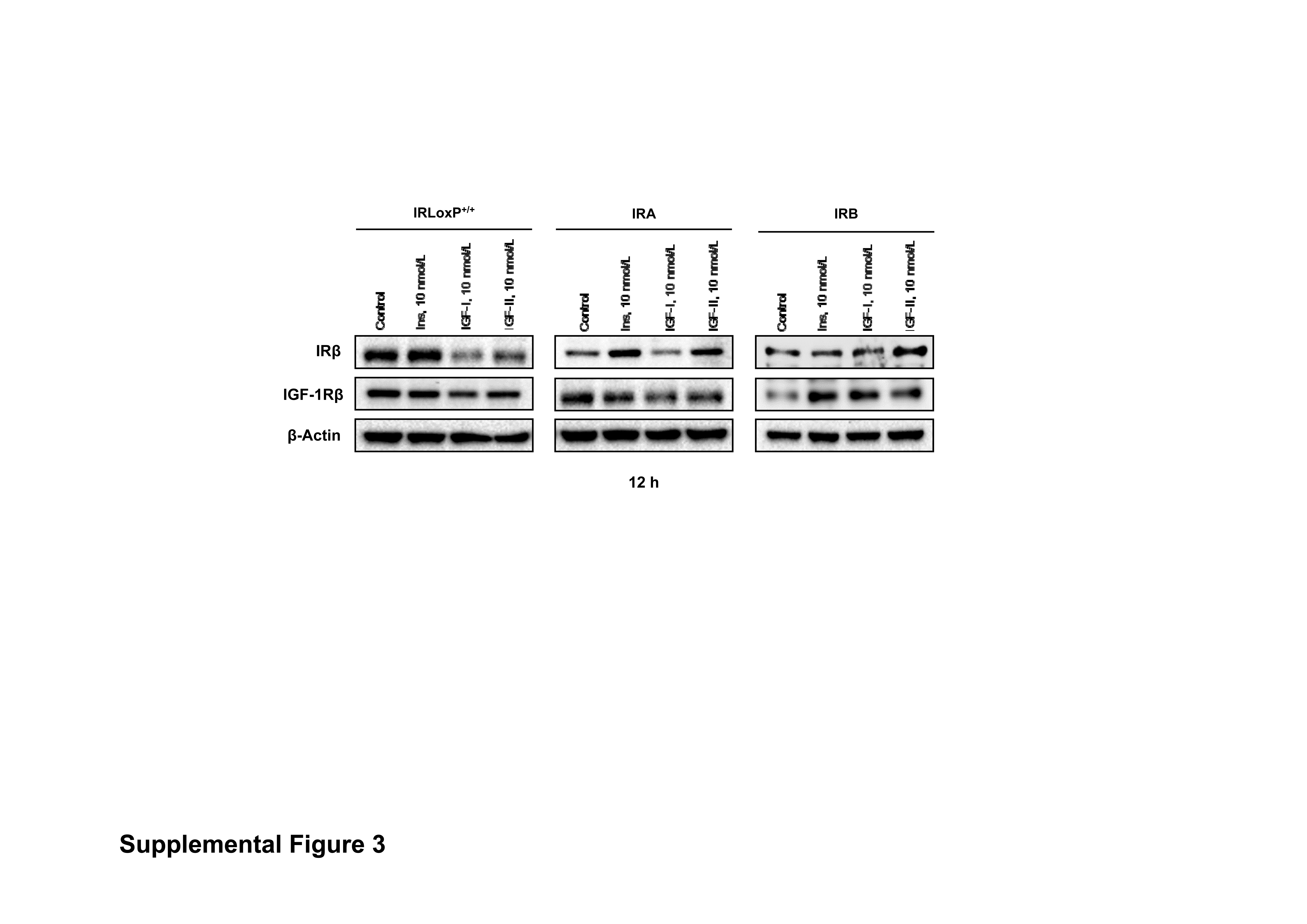

Supplement: Supplementary file 3 — Additional file 3. Western blot analysis of IR and IGF-IR expression in IRLoxP+/+, IRA and IRB VSMCs stimulated with insulin, IGF-I or IGF-II for 12h. [file 12933_2016_477_MOESM3_ESM.tif]

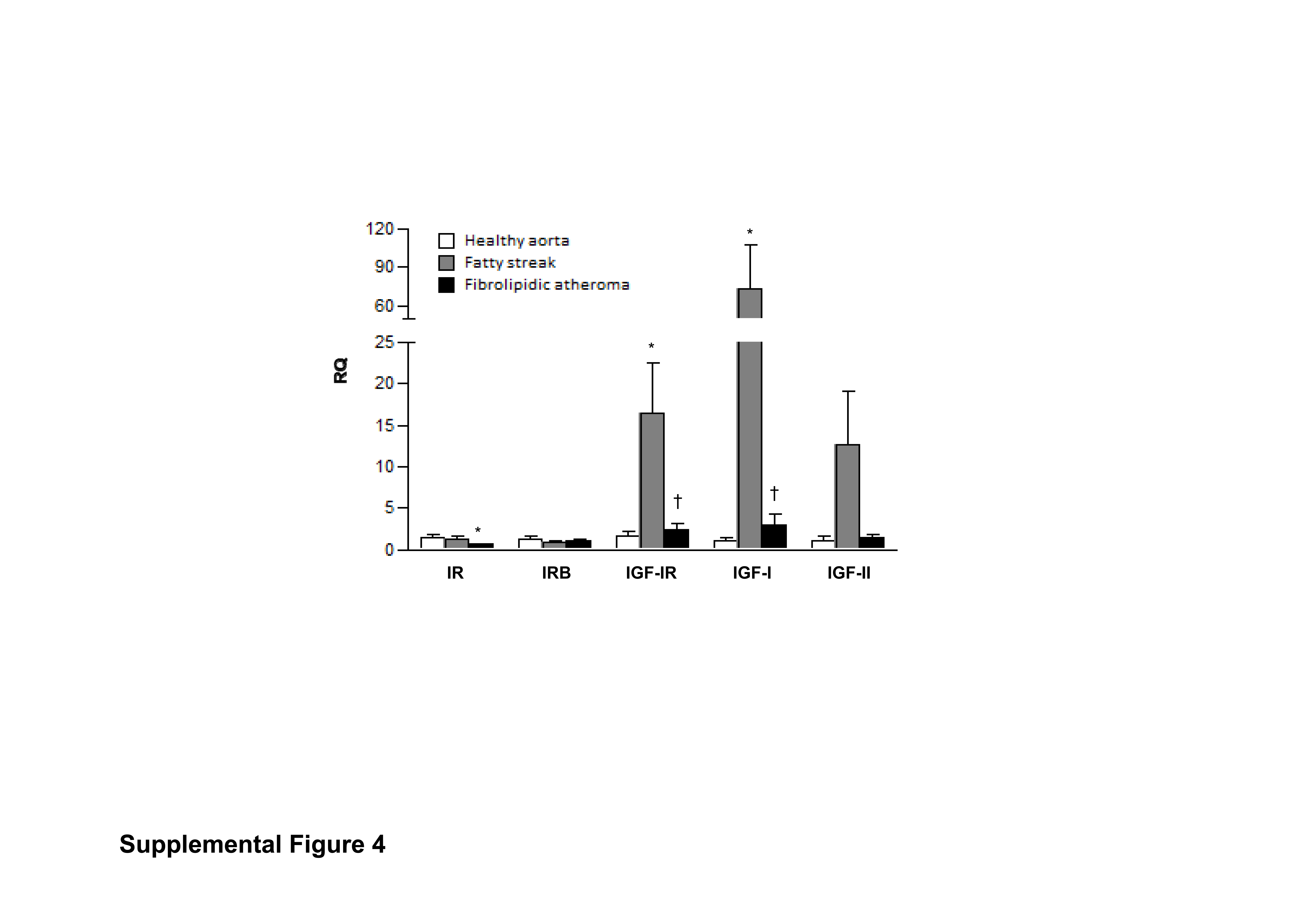

Supplement: Supplementary file 4 — Additional file 4. Increased expression of IGF-IR and IGFs in media from human aortas bearing fatty streaks. Analysis of mRNA expression of total IR, IRB isoform, IGF-IR and IGFs by qRT-PCR in the medial layer of 28 human aortas (8 healthy, 9 fatty streaks and 11 fibrolipidic lesions). *p<0.05 vs. healthy aorta; †p<0.05 vs. fatty streak. [file 12933_2016_477_MOESM4_ESM.tif]
